# Mental Health and Work-Related Behaviors in Management of Work Requirements of University Lecturers in Ukraine—An Age Group Comparison

**DOI:** 10.3390/ijerph182010573

**Published:** 2021-10-09

**Authors:** Beatrice Thielmann, Håvard Rudi Karlsen, Myroslav Tymbota, Valeriy Kapustnyk, Nathalie Zavgorodnia, Igor Zavgorodnii, Irina Böckelmann

**Affiliations:** 1 Institute of Occupational Medicine, Medical Faculty, Otto-von-Guericke-University, 39120 Magdeburg, Germany; irina.boeckelmann@med.ovgu.de; 2 Department of Psychology, Norwegian University of Science and Technology, 7491 Trondheim, Norway; h.karlsen@ntnu.no; 3 Educational and Scientific Institute for the Education, Kharkiv National Medical University, Kharkiv, Ukraine; wal.bale98@gmail.com (M.T.); zavnikua@gmail.com (I.Z.); 4 Kharkiv National Medical University, Kharkiv, Ukraine; kapustnik.valerij@ukr.net; 5 Department of Neurosurgery, Internal and Occupational Diseases, Kharkiv National Medical University, Kharkiv, Ukraine; z.nataliia@gmail.com

**Keywords:** work-related behavior and experience patterns (AVEM), general health questionnaire (GHQ-12), mental health, prevention, university lecturers, stress

## Abstract

Background: There are only a few national and international studies on the health of third-level professor lecturers at universities and colleges. Work-related diseases are important and relevant for occupational medicine. The aim of the study was to examine the mental health and work-related behavior and experience patterns of Ukrainian university lecturers in age group comparisons. Methods: Data were collected from 81 Ukrainian university lecturers (General Health Questionnaire (GHQ-12), Questionnaire on Work-Related Behavior and Experience Patterns (AVEM)). The university lecturers were split into 4 age groups. Results: 9.9% of higher education lecturers reported impaired mental health (GHQ-12). In all, 64.8% of the total sample showed AVEM risk patterns. There were differences in age groups (third age group scoring lower than the first age group on the GHQ total score). The first age group had significantly lower opinions of the subjective importance of work on their personal lives compared to the third and fourth age group, while the second age group had significantly lower opinions compared to the fourth age group. All individuals with impaired mental health in GHQ-12 had AVEM risk patterns. Conclusions: These results offer novel insights into the health of Ukrainian university lecturers. Occupational healthcare practitioners should take them into consideration in order to initiate appropriate interventions.

## 1. Introduction

The health of teachers has been well examined in national and international studies. In contrast, the health of university lecturers is rarely studied in international studies. 

Working conditions at universities are a topic of much debate, though data on university lecturers from Ukraine in particular are scarce. On one hand, physical stress factors affecting university lecturers such as noise or vocal stress are important to consider [1]. On the other hand, there are also various high mental stress factors such, as communication between students, other lecturers or supervisors, conflicts with colleagues, administrative problems, and lack of recognition of one’s own work by colleagues, supervisors or students [2]. Temporary contracts can be an additional burden and make academic development and family planning uncertain. In addition to teaching, university lecturers are also involved in other fields of activity, such as the acquisition and management of third-party funding, implementation and supervision of research studies, leadership in project groups, training of project officers in methodology, publication activities, and supervision of graduation work (bachelor’s and master’s theses or doctoral dissertations), as well as various tasks in the form of volunteer work in various university committees or as reviewers of publications in journals or research applications or graduation work. Research has shown that Ukrainian university lecturers face the main stressors of conflicts, poor support from colleagues or school management, and dissatisfaction with the professional position and climate at the university [3]. It is important to make a distinction between university lecturers educating at universities and colleges and whether there is a focus on teaching, research, or both. Working at a university clinic includes additional burdens, such as providing healthcare. A study suggests that clinical experience is key to learning processes by, for instance, providing a relevant context for understanding and promoting memory in medical teachers [4]. New quality requirements for universities and colleges are in favor of hiring new lecturers with a focus on teaching, as this results in an improvement in the quality of teaching. Under constant national and international competitive pressure, which is increasingly noticeable at colleges, an increasing trend for combining teaching and research can be also observed at colleges. In a recent study, university professors had a more positive view of their own educational activities compared to professors at colleges or other staff [5]. The reasons for this are the support of their own research and that of young scientists [5]. Results from German universities showed that employment terms have no influence on the satisfaction of teaching, but the present high work autonomy has a positive influence on job satisfaction [5]. In recent years, pay incentives and symbolic motive incentives have been increasingly popular in Germany to further develop or improve teaching. There are performance incentives in the form of, e.g., appeal payment, salaries for special benefits, and location-based defined performance-based resources allocation, target agreements, and teaching awards. In addition, the use of temporary contracts makes academic development and family planning uncertain. Nevertheless, temporary employees seem to be more effective at their jobs than those with permanent jobs.

Work-related mental stress (for example, high workload and low reward) can cause, for instance, cardiovascular diseases [6], anxiety disorders [7], or depression [8]. If high workloads are not adequately managed, people may develop unhealthy patterns of behavior and experience and also exhibit burnout symptoms [9].

According to the German Absenteeism Report 2018, the lowest sick leave rates were seen for academic occupational groups (e.g., university teaching and research, doctors). We were unable to retrieve any such data from Ukraine. Tymbota et al. [2] showed that younger university lecturers up to the age of 35 years have unfavorable burnout dimensions, and there were significant differences in the burnout dimensions of “emotional exhaustion” and “cynicism”. Health-promoting or unhealthy attitudes and habits in work management are recorded in the Questionnaire on Work-Related Behavior and Experience Patterns (AVEM) [10]. In all, 40% of 481 German teacher students exhibited unhealthy work-related behavior and experience patterns during their studies [11]. In an occupational group comparison between nurses, bank employees, and medical assistants, teachers exhibited the highest intrinsic overcommitment. Age group differences were found for overcommitment (increased with age). Additionally, 4.8% of the teachers in this study also presented with serious burnout [12]. A study of teachers found a slight decrease in mental performance associated with age, especially in the area of fluid components. Fluid components describe, e.g., speed and precision of information processing and perception in people [13]. Mental performance is a major resource for coping with workloads and successful aging [14]. Decrease in such performance can result in reduced wellbeing in old age and have an adverse effect on mental health [13]. The requirements for lecturers are different in each country, and the average teaching load in Ukraine depends on the position of employment, while the load constitutes the following number of work hours: for assistants, 600 h yearly; for associate professors, 560 h yearly; and for professors, 520 h yearly. In Germany, the teaching load depends, e.g., on the type of university. At university, it is 224 h yearly, and at local colleges, 504 h yearly. Regarding promotion opportunities in Ukraine, the following criteria are assumed and are comparable to those found in other countries: defense of dissertation (for obtaining Ph.D. status), permanent professional development (including participating in scientific international educational events and fellowships), as well as certified, highly proficient knowledge of the English language.

In addition to the high workload, university teaching provides room for personal growth, which can counteract stress. The didactic training of all lecturers is usually only a university standard in countries such as the USA or the United Kingdom [15]. Optional training in higher education didactics for teachers is often offered. Lecturers’ leadership behaviors have been linked to better university teaching [16]. Leadership behavior is trainable and can increase feelings of personal development.

There are hardly any data available at present on Ukrainian university lecturers. To involve Ukrainian university lecturers in international comparisons, the aim of the present survey was to examine the mental health and work-related behavior and experience patterns of Ukrainian university lecturers through age group comparisons. By doing this, we hope to expand the knowledge around university lecturers in Ukraine and allow for more research on them and on university lecturers internationally. Additionally, it is possible to identify mental stress at an earlier stage as a first step of prevention, whereby hazards to health could be reduced. The results of this study can help to develop a more sustainable work environment for Ukrainian university lecturers. We hypothesized that an age dependency by mental health and AVEM exists in favor of younger subjects.

## 2. Materials and Methods

Data from 81 university lecturers from a Ukrainian higher medical educational institution were taken into account. The mean age was 48.5 ± 13.2 years. The study was conducted between January 2016 and December 2017. Subjects were invited to participate in the study through noticeboards and personal messages. Participants were selected randomly and in an equal proportion from clinical, medical–biological, as well social–economical departments in order to equitably present all the above mentioned fields of scientific activity. Participants were invited to book an appointment to collect their questionnaire. The return deadline was 2 days.

Data from the following questionnaires were collected in paper form along with the age of participants:

- General Health Questionnaire (GHQ-12) by Goldberg [17]; and
- Work-Related Behavior and Experience Patterns (AVEM) by Schaarschmidt and Fischer [10].

### 2.1. General Health Questionnaire (GHQ-12) 

The GHQ-12 by Goldberg [17] records short-term changes in the state of health. There is a German version of the GHQ-12, developed by Linden et al. [18]. There is also a validated, standardized version in Russian for the GHQ-12 [19]. This was also used in Ukraine, since all participants spoke Russian. Symptoms such as anxiety, sleep disturbances, or physical discomfort from the last 14 days are registered in this questionnaire. The self-administered questionnaire collects a total of 12 items (scored 0-0-1-1). The answer options are: “not at all”, “not more than usual”, “worse than usual”, or “much worse than usual”. The GHQ-12 shows good reliability (α = 0.85) and homogeneity (interitem correlation r = 0.40–0.65).

The evaluation and classification of the total score of GHQ-12 was carried out according to Goldberg and Williams [17]:

- GHQ total score ≤ 4.0 corresponds to regular mental health (considered here group G0);
- GHQ total ≥ 5.0 corresponds to impaired mental health (group G1).

### 2.2. Work-Related Behavior and Experience Patterns

The Questionnaire on Work-Related Behavior and Experience Patterns by Schaarschmidt and Fischer [10] includes 66 statements regarding health-promoting or -impeding attitudes and habits in work management. For the AVEM, there are official translations to Russian in the standardized test battery of the Vienna Test System (Schuhfried GmbH, Austria). These combine into the areas of “work engagement”, “resilience to stress”, and “emotions” across the 11 following dimensions: (1) subjective importance of work in personal life, (2) work-related ambition, (3) willingness to work until exhausted, (4) striving for perfection, (5) distancing ability (ability to recuperate mentally from work), (6) tendency toward resignation in the face of failure, (7) proactive problem solving (active and optimistic attitude), (8) inner calm and balance (experience of emotional stability), (9) experience of success at work (satisfaction), (10) satisfaction with life, and (11) experience of social support. 

This enables participants to be assigned based on a certain probability to one of a number of patterns. The description of the four different patterns follows: 

Pattern G: Strong work-related ambition, existing distancing ability, high resistance, low tendency to resignation, proactive problem solving, positive attitude to life;

Pattern S: Occupational protection, low work-related ambition and low willingness to work until exhausted, high distancing ability, high level of inner calm and balance, positive attitude to life (outside work);

Risk Pattern A: Highest subjective importance of work in personal life, expanded work engagement, striving for perfection and willingness to work until exhausted at first line, low level of inner calm and balance, poor resilience to stress. The pathogenic impact factor is seen in the combination of the described behavior pattern with negative feelings. The result is a personality which increases, for example, the risk of cardiovascular diseases [10];

Risk Pattern B: Low level of work engagement, subjective importance of work in personal life, limited distancing ability, highest tendency toward resignation in the face of failure, low level of satisfaction with life and experience of social support. There is a bundle of emotional impairments with general feelings of exhaustion, associated with feelings of hopelessness and feeling of depression. This does not mean that every case necessarily progresses from a state of “burning” to “burnout” (pathway Risk Pattern A to Risk Pattern B) [10].

The participants’ answers to the 11 subscales were combined using weights. These were based on validated cluster analysis performed on the AVEM (12). Each participant was then delegated to one of four AVEM patterns, based on the amount of correspondence between each person’s answers and the patterns. These were those that corresponded either to a pure pattern with at least 95% expression, an accentuated pattern with a manifestation between 81% and 94% in a pattern, or a trend characteristic between 50% and 80% in a pattern. Mixed patterns were not considered. The reliabilities (Cronbach’s α) of the different scales ranged from 0.81 to 0.86.

### 2.3. Statistical Methods

All calculations were carried out with SPSS 24.0. The levels of significance were set at 5% (*p* < 0.05 significant, *p* < 0.01 very significant, and *p* < 0.001 most significant) for all studied parameters. Initially, we checked the variables for normal distribution using the Kolmogorov–Smirnov test. This was followed by a descriptive and explorative analysis of the total sample. The tests between the two categorical variables of GHQ score and AVEM pattern were implemented using Fisher’s exact test. Bonferroni corrections were made for multiple comparisons. Comparisons between the four age groups were made using one-factorial analysis of variance (ANOVA) followed by a Bonferroni post-hoc test. To account for non-normal distributions of the variables, Spearman’s correlation coefficient rho was calculated for the relationship of all the AVEM main variables with GHQ and age, respectively. The evaluation of effect size was informed by Cohen, with rho < 0.10 corresponding to a weak effect, rho < 0.30 to a medium effect, and rho ≥ 0.50 to a strong effect.

## 3. Results

### 3.1. Age Group Differences

The mean age of the 57 women (70%) and 24 men (30%) was 48.5 ± 13.2 years, and the mean years at work were 19.0 ± 12.6 years. The weekly working hours were 35.8 ± 11.7 h per week. Most employment contracts (76%) are limited to working hours of 35.8 h. 

To ensure a relatively even distribution of participants in the age groups, participants were divided into age groups (AGs) according to four quartiles of age (see Table 1).

### 3.2. Allocation of GHQ Groups within Age Groups

**Figure 1:**
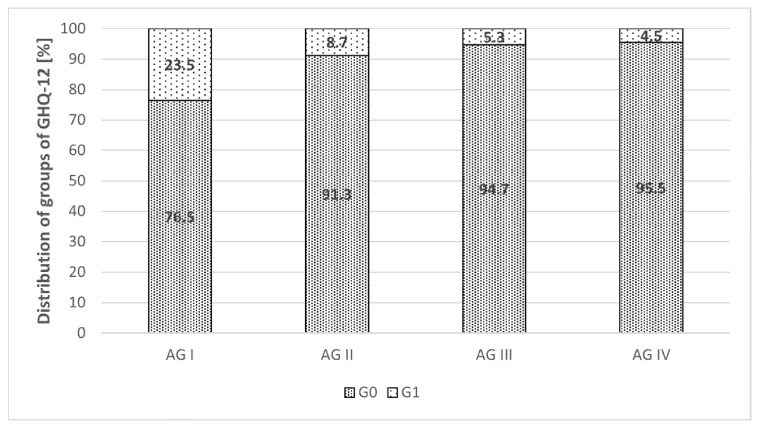
Distribution of groups of GHQ-12. Notes. AG = age group G0 = group with regular mental health, G1 = group with impaired mental health.

An impaired mental health by GHQ-12 (G1) was determined in 9.9% (*n* = 8) of university lecturers. Of these, 50% (*n* = 4) were represented in AG I, 25% (*n* = 2) in AG 2, and 12.5% (*n* = 1) each to AG III and IV. The distribution is shown in Figure 1. There were no significant differences.

### 3.3. Allocation of GHO Total Score within Age Groups

The lowest mean score of GHQ total score was 8.3 ± 4.2 for AG III. The highest mean score was registered for AG I with 12.1 ± 4.5, which was significantly different from AG III (*p* = 0.019). The results are shown in Table 2.

### 3.4. Allocation of AVEM Groups within Age Groups

**Figure 2:**
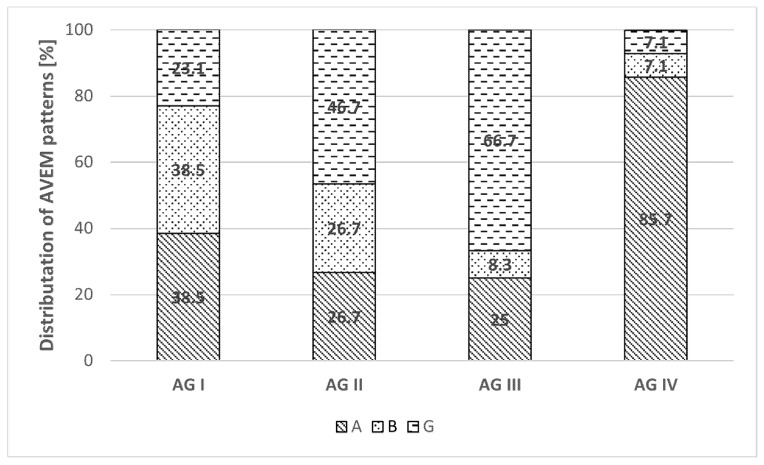
Allocation of AVEM patterns A, B and G within age groups.

In total, 66.7% (*n* = 54) of university lecturers were determined to belong to a pure, an accentuated or a trend characteristic pattern. Figure 2 shows the distribution within age groups. In all, 64.8% (*n* = 35) of these people had to be assigned to AVEM risk patterns A or B. Only 35.2% (*n* = 19) of university lecturers could be assigned to one of the two health-promoting AVEM patterns. This was exclusively AVEM pattern G. The AVEM S pattern was not present at all. A particularly high proportion of AVEM risk patterns A and B was represented in AG IV with 92.8% and AG I with 77.0%. Only in AG III was the predominant health-promoting AVEM pattern G found.

All 8 participants with impaired mental health exhibited AVEM risk patterns. Of these, 37.5% (*n* = 3) were determined to belong to AVEM risk group A and 62.5% (*n* = 5) to AVEM risk group B.

### 3.5. Comparison of AVEM Dimensions between Age Groups

The stanine values of individual AVEM dimensions of university lecturers were in the normal range (stanine 3.5–6.6 points) according to the AVEM manual [10], whereby “tendency toward resignation in face of failure” was at the lower standard limit in AG III. The AVEM main variable “subjective importance of work in personal life” was the lowest in AG I, significantly different from AG III (*p* = 0.01) and AG IV (*p* < 0.001). AG II also scored significantly lower than AG IV (*p* = 0.034). Furthermore, the “tendency toward resignation in face of failure” was the lowest in AG III, which was significantly different compared to AG I and II (*p* = 0.044) and to AG IV (*p* = 0.002). The results are shown in Table 3.

### 3.6. Correlations between AVEM Dimensions, GHQ Total Score, and Age Groups

The results of the Spearman correlation analysis between AVEM dimensions and the GHQ-12 total score or age are shown in Table 4. There was a moderate positive correlation between “tendency to resignation in the face of failure” (rho = 0.496, *p* < 0.001) and GHQ-12 total score. Moderate negative correlations were found between GHQ total score and AVEM dimensions “inner calm and balance (experience of emotional stability)” (rho = −0.449), “experience of success at work (satisfaction)” (rho = −0.408), and “satisfaction with life” (rho = −0.469); each *p* < 0.001. With regard to age, there was only a high positive correlation for the dimension “subjective importance of work in personal life” (rho = 0.520, *p* < 0.001).

## 4. Discussion

The study shows that a minor proportion of university lecturers reported impaired mental health, and the majority of the subjects had AVEM risk patterns (all lecturers with impaired mental health in GHQ-12 had AVEM risk patterns). There were differences between age groups in GHQ total scores, subjective importance of work in personal life, and tendency toward resignation in the face of failure. 

In contrast to the health of teachers, which is well studied around the world, the health of lecturers is less well studied, and there are hardly any data on Ukrainian university teachers in particular. The available data from the health status of the Ukraine university lecturers of different faculties of a university offer important and novel insights. 

The workload of the profession is varied. In addition to teaching and research, other examples for lecturers are also supervision tasks for students, and administrative tasks such as quality assurance. As a result, university lecturers see themselves as service providers, with less time remaining for research. On the other hand, work-related autonomy, social integration, and competence experience in the sense of self-determination theory, summarized as basic human needs according to Deci et al. [20], are positively evaluated. 

Nearly 10% of the university lecturers in the current sample exhibited impaired mental health (G1) according to the GHQ-12 (GHQ total ≥ 5.0). Likewise, the GHQ total score was highest in AG I and lowest in AG III. This is slightly more than for Ukrainian bank employees, 8.8% of whom have impaired mental health [21]. However, this value is not so high as in other occupational groups in other countries. Data from the GHQ-12 on lecturers in other countries are limited. A study in Germany found impaired mental health in 25.6% of medical assistants, 38.8% of nurses, 20% of bank employees, and 24.7% of schoolteachers [12]. Another study also found impaired mental health in 25% of Italian schoolteachers [22]. University lecturers thus seem to have better mental health than teachers.

In 64.8% of the total sample, AVEM risk patterns A or B were determined, which is an alarming finding. According to the AVEM manual, this indicates that those belonging to those patterns have problems emotionally distancing themselves from their work, or they are at risk of burnout or cardiovascular diseases [10]. Only 35.2% of the total sample was grouped into health-promoting AVEM pattern G. AVEM pattern S was not represented at all. In comparison, among Ukrainian bank employees, 42.2% had health-promoting AVEM patterns [23], and the sample used in that study was of a similar size to the present sample of lecturers. Overall, 92.8% of the participants in AG IV belonged to risk patterns A or B, and 77.0% of participants in AG I belonged to risk patterns A or B. Data from AVEM on lecturers in other countries are limited. Compared to other occupational groups in Germany, the AVEM risk groups of Ukrainian lecturers can be classified as higher than those for 45.7% of businessmen, 56% of teachers, and 44.1% of physicians [24]. 

Those with AVEM risk patterns need intervention, e.g., coaching on self-management. The individual consideration of the AVEM dimensions revealed significant age group differences, but only in the variables “subjective importance of work in personal life” and “tendency toward resignation in the face of failure”. Additionally, there are moderate positive relationships between GHQ total score or age and the AVEM dimension “subjective importance of work in personal life". Our results showed that people with a lower degree of mental health tended to a have a lower value in “inner calm and balance (experience of emotional stability)”, “experience of success at work (satisfaction)”, and “satisfaction with life”. Our own research also showed that this age group, up to 35.5 years of age, presented less favorable burnout dimensions and significant differences in the dimensions of “emotional exhaustion” and “cynicism” [2]. Another study found out that 1/3 of female university graduates were overworked and showed resignation tendencies [25]. Additionally, the GHQ total score of female Japanese lecturers was found to be significantly higher than that of males [26]. Often-neglected relations between lecturers and students play an important role in university teachers’ perceptions of exhaustion and engagement. Similarly, mental health was impaired in 31.3% of German student teachers [27], while a study on German bank employees showed an impaired mental health among 23% of the subjects [28], and another on emergency physicians found the same for 16.1% of the participants [29]. 

In this sample, younger lecturers showed higher mental impairment and less favorable AVEM patterns. Overcommitment appears to be elevated at younger ages because young people are at the beginning of their professional career. They deal with science and with their dissertations. In addition, they have to cope with fixed-term employment agreements. As many people are engaged in family planning during this phase of their life, the lower subjective importance of work in personal life in our sample seems plausible. A comparison of age groups showed significant differences between teachers under 30 or 50 years of age compared to elderly teachers [12]. Younger lecturers show here the lowest subjective importance of work in personal life and a tendency toward resignation in the face of failure. To mitigate this, appropriate training in university didactics could be offered to lecturers. Improved methodology in terms of personal development could also improve the subjective importance of the work. The subjective importance of work in personal life correlated negatively with mental health in the GHQ-12. Therefore, an improvement in the importance of work could be associated with improved mental health. This should be investigated in longitudinal studies.

These results should be taken into account in occupational healthcare. In addition to behavioral prevention as listed above, a relationship-based prevention should be added. According to the manual [10], the targets for interventions for AVEM risk patterns are to be found in several areas, as well as in life overall. Workplace inspections should be carried out. Furthermore, the authors of the AVEM manual recommend that the quantity of work demands, individual organization of work, time management, and social relationships in the workplace be considered and evaluated [10]. A reduction in external stress should be a top priority in primary prevention approaches. Involving the employees in decisions, having regular team meetings, supervising, and showing appreciation for work carried out are important factors which have a positive impact on employees. A study on 150 Australian health and safety officers showed that an increase in health and safety in the workplace makes a positive contribution to working relationships [30]. A Dutch study indicated that an increase in worker participation correlates with better quality risk assessments and more preventive measures against psychological strain [31]. Mentoring relationships were identified by students and lecturers as motivating or hindering the educational experience from both a personal and professional perspective [32]. Mentorship or supervision should be integrated in health prevention programs to increase levels of job satisfaction and feelings of job control in the workplace [25].

A prevention team of occupational psychologists and health economists under the direction of a physician of occupational medicine would be useful for effective prevention in companies. Vulnerable workers should be given special care. Such workers could be detected using the same questionnaires as we used here. National occupational health policies are directed to the identification of prepathological conditions in academic lecturers (including schoolteachers), which can lead to professional burnout syndrome. What is more, factors that could have a great influence on the health of teachers should be considered. Great importance is also assigned to the necessary selection criteria of people involved in the teaching process. National occupational health policies suggest standardization and rationing of the work of academic teachers, with the aim to protect and maintain their health. Currently, health policy in Ukraine is undergoing major changes, including in the field of occupational medicine.

### Limitations

One limitation of this study is the initial use of the questionnaire in paper format, which could guarantee the anonymity of the participants to a lesser degree than, for instance, an online questionnaire. We did not assess or try to detect social desirability and thus cannot exclude it. Another limitation of this study is that we only used pure pattern, accentuated pattern, and pattern with trend characteristics of the AVEM. Mixed patterns were not considered here. In addition, it is not certain that the data on Ukrainian higher education lecturers are transferable to lecturers of other nationalities. Newer teaching methods such as digital distance learning were not considered because data were collected before the COVID-19 pandemic. Studies show both positive and negative consequences of digital distance learning at universities [33]. The sample is small and is not representative (voluntary participation). The possibility of a selection bias toward participation by more psychologically affected persons cannot be excluded. A higher sample size for future studies is thus recommended. The correlation of the instruments with other variables (e.g., small, medium, and large universities) or the application of the instruments on population groups from other disciplines (e.g., technical, sociohuman), could lead to more relevant results in the local context. These could be the subject of analysis of future research for authors. Only two psychological tools were used in the study. In future research, organizational variables such as organizational support and workload are also worth considering. However, this is also a strength of occupational medicine because it meets “seemingly healthy people” who do not go to the general practitioner because of anxiety or shame, for example [34].

## 5. Conclusions

In this sample of Ukrainian university lecturers, all participants with impaired mental health also have risk patterns in work-related behavior and experience patterns. We found age-related differences in general mental health. The youngest lecturers (age up to 35.5 years) had the highest GHQ total score in comparison to the lowest score of age group III (48.6–56.6 years). Occupational healthcare practitioners should take them into consideration in order to initiate appropriate interventions. The importance and necessity of this research for occupational health programs in a national and international context are great in view of keeping employees healthy in the long term. Healthy university faculty are more productive and competitive, even from a perspective of educating students on a national or international career path. The results of this study can help us to create a more supportive work environment for lecturers. This could allow people to be recruited to the profession and provide encouragement for existing teachers to stay in the profession.

## Figures and Tables

**Table 1:** Allocation of age groups.

Age Group	*n*	AV ± SD (Years)	Range (Years)
AG I	17	31.6 ± 3.5	23.6–35.5
AG II	23	47.6 ± 3.7	36.6–47.6
AG III	19	52.8 ± 2.7	48.6–56.6
AG IV	22	65.2 ± 6.4	58.6–78.6

**Table 2:** GHQ total score in each age group (AV ± SD = average and standard deviation, minimum/maximum value).

Age Group	AV ± SD	Min	Max	*p*_ANOVA_	*p*_Bonferroni_
AG I	12.1 ± 4.5	5	19	* 0.024	AG I/III 0.019
AG II	10.9 ± 3.1	5	17		
AG III	8.3 ± 4.2	4	21		
**AG IV**	**10.3 ± 3.3**	**6**	**18**		

Note: * *p* < 0.05. Age groups: AG I = 23.6 to 35.5 years, AG II = 36.6 to 47.6 years, AG III = 48.6 to 56.6 years, AG IV = 22; 58.6 to 78.6 years.

**Table 3:** Characteristics of AVEM dimensions taking into account age groups (Stanine M ± SD = mean and standard deviation).

AVEM Main Variables	AG I	AG II	AG III	AG IV	Total	*p*_ANOVA_	*p*_Bonferroni_
Subjective importance of work in personal life	5.0 ± 1.97	6.0 ± 1.22	6.6 ± 1.17	7.2 ± 1.40	6.2 ± 1.62	<0.001	I/III 0.010 I/IV < 0.001 II/IV 0.034
Work-related ambition	6.7 ± 1.31	6.8 ± 1.04	6.3 ± 1.24	6.3 ± 1.46	6.5 ± 1.27	0.447	n. s.
Willingness to work until exhausted	5.3 ± 1.90	6.0 ± 1.02	5.4 ± 1.61	6.0 ± 1.53	5.7 ± 1.52	0.339	n. s.
Striving for perfection	5.2 ± 2.02	6.1 ± 1.38	5.9 ± 1.45	5.8 ± 1.63	5.8 ± 1.61	0.418	n. s.
Distancing ability (ability to recuperate mentally from work)	4.9 ± 1.83	6.4 ± 8.28	5.7 ± 1.46	4.5 ± 0.91	5.4 ± 4.57	0.498	n. s.
Tendency toward resignation in the face of failure	4.9 ± 1.36	4.8 ± 1.80	3.5 ± 1.50	5.3 ± 1.36	4.6 ± 1.65	0.002	I/III 0.044 II/III 0.044 III/IV 0.002
Proactive problem solving (active and optimistic attitude)	4.7 ± 1.86	4.7 ± 2.18	5.4 ± 1.92	4.9 ± 1.78	4.9 ± 1.93	0.678	n. s.
Inner calm and balance (experience of emotional stability)	4.7 ± 1.37	5.1 ± 1.56	5.6 ± 1.84	4.6 ± 1.36	5.0 ± 1.56	0.192	n. s.
Experience of success at work (satisfaction)	4.4 ± 2.00	5.1 ± 2.00	5.5 ± 1.61	5.1 ± 1.43	5.0 ± 1.78	0.317	n. s.
Satisfaction with life	4.1 ± 1.73	4.6 ± 1.70	4.6 ± 1.34	4.5 ± 1.50	4.5 ± 1.59	0.746	n. s.
Experience of social support	4.5 ± 1.74	4.4 ± 1.75	5.2 ± 1.61	4.2 ± 1.88	4.6 ± 1.76	0.172	n. s.

Commentary: n. s. = not significant, AG = age groups. stanine normal range 3.5 to 6.5, age groups: AG I = 23.6 to 35.5 years, AG II = 36.6 to 47.6 years, AG III = 48.6 to 56.6 years, AG IV = 22; 58.6 to 78.6 years.

**Table 4:** Correlations (Spearman’s rho) between AVEM main variables, GHQ-12 sum total, and age.

AVEM Main Variables	GHQ Sum Total	Age
Subjective importance of work in personal life	−0.282 *	0.520 **
Work-related ambition	−0.051	−0.144
Willingness to work until exhausted	0.121	0.084
Striving for perfection	−0.282 *	0.095
Distancing ability (ability to recuperate mentally from work)	−0.068	0.013
Tendency toward resignation in the face of failure	0.496 **	−0.007
Proactive problem solving (active and optimistic attitude)	−0.272 *	0.102
Inner calm and balance (experience of emotional stability)	−0.449 **	−0.033
Experience of success at work (satisfaction)	−0.408 **	0.154
Satisfaction with life	−0.469 **	0.087
Experience of social support	−0.271 *	−0.005

Note: * *p* < 0.05, ** *p* < 0.01.

## Data Availability

The data can be accessed via the corresponding author. They are archived at the University of Magdeburg.
